# The delay in the diagnosis and treatment of newly diagnosed bladder cancer patients during COVID 19 pandemic

**DOI:** 10.4314/ahs.v22i3.26

**Published:** 2022-09

**Authors:** Cagri Dogan, Cenk Murat Yazici, Haci Murat Akgül, Onder Cinar, Hüseyin Ateş, İlyas Yaz

**Affiliations:** 1 Namik Kemal University Faculty of Medicine, Department of Urology; 2 Zonguldak Bulent Ecevit Universitesi Tip Fakultesi; 3 Zonguldak Bulent Ecevit Universitesi Tip Fakultesi

**Keywords:** COVİD-19, bladder cancer, pandemic, treatment

## Abstract

**Background:**

To evaluate the newly diagnosed bladder cancer(BC) patients during the pandemic period and compare them with the corresponding last4 years.

**Objectives:**

To document the time schedules of BC patient evaluation and define the possible delays and investigate the reasons.

**Methods:**

Newly diagnosed BC patients who underwent transurethral resection of bladder tumour in the last5 years were retrospectively included to study. The patients were divided into 5 groups. Group-1 was composed of patients diagnosed betweenMarch, 1,2016 -March-1,2017. The patients who were diagnosed in the further corresponding years formed group 2,3 and 4. The last group(Group-5) was composed of patients who were diagnosed during the pandemic period which was between March,1,2020 and March,1,2021. The clinicodemographic properties and diagnostic time schedules of the patients were compared between the groups.

**Results:**

There were56 patients in Group-1,60 patients in Group-2,61 patients in Group-3,68 patients in Group-4, and 58 patients inGroup-5. The mean hospital admission period was102.5±179.0days during the pandemic period which ranged between24.5± 32.0 and38.3±69.1days before thepandemic.(p=0.002)The diagnosis-anesthesia period was significantly higher during the pandemic pandemic period.(p=0.034)

**Conclusions:**

The pandemic period has caused some delays in the diagnosis and treatment of BC patients. Telemonitoring systems may be useful to prevent the possible diagnostic and treatment delays for newly diagnosed BC patients.

## Introduction

Bladder cancer (BC) is the 10th most common cancer in the world with high mortality rates 1 Early diagnosis of BC is significantly important to decrease cancer-specific mortality. Nearly 75% of the BC patients are diagnosed with the noninvasive disease and can be treated by organ sparing modalities with strict surveillance.^2^,^3^ On the other hand, any delay in the diagnosis and the surveillance may lead to the progression of BC and may lead to morbid and mortal outcomes. It was shown that a delay of radical cystectomy more than 12 weeks after the diagnosis of muscle-invasive bladder cancer (MIBC) significantly interfered with the life expectancy of the patients.^4^ Even the immediate radical cystectomy for high-risk non-muscle invasive bladder cancer (NMIBC) was superior to deferred radical cystectomy in terms of cancer-specific survival.^5^ These data document the importance of early diagnosis and timely treatment of BC.

The new severe acute respiratory syndrome SARS COV-2 is a life-threatening infectious disease that was declared a pandemic by the World Health Organization (WHO) on March 10, 2020.^6^ It is a highly transmissible disease that may cause severe respiratory dysfunction and even death. This pandemic incredibly affected social life and the governments had to take significant precautions to manage the outbreak. The educational system was affected as well as the healthcare system due to strict restrictions and face to face classical education model could not have been maintained. Governments had to alternate the classical education system to online and telemonitoring-based systems. Some departments were more seriously affected than others especially surgical residents who had to learn surgical procedures face to face in their clinics. Busetto et al. reported that the COVID-19 pandemic affected urology residents' clinical and learning activities, and face-to-face training programs for surgery were in a dramatic decline in terms of quantity and quality. In the same study, they observed that although the Italian health care system and residence educational program is the third-best in the world, they faced a dramatic change in residents' daily routines in a decrease in all clinical and learning activities.^7^–^9^

The first case was observed on March 11 in our country and the government immediately started the preventive measures. The public activity was stopped with strict restrictions till to June 1, 2020. During the summertime, the restrictions were loosened with precaution but the elderly population older than 65 years of age were still under strict restrictions. The second wave of the pandemic was observed after the summer and new restrictions were re-scheduled at the end of October.^10^ During the restriction period, both the public and private hospitals were re-organized to deal with the pandemic. The elective surgeries were postponed and the daily activity of outpatient clinics was limited. Our institution as a tertiary center allowed the only emergency and oncological surgeries and a telemonitoring outpatient clinic system was activated. The surgical procedures for bladder cancer (BC) were classified as oncological surgeries and were performed according to risk categorization. Including the patients with BC, this unusual period might cause some difficulties for the patients to reach the clinicians. In a study, De Vincentiis et al reported a 66% decrease in the diagnosis of BC during the pandemic period.^11^ We believe that documenting the possible delays for the diagnosis of BC and evaluate the reasons for these delays is significantly important.

The primary aim of our study was to evaluate the newly diagnosed BC patients during the pandemic period and compare them with the corresponding past 4 years. The secondary aim was to evaluate the possible delays during the time schedules of BC patients starting from the day of initial symptom to the day of the pathology report. The last aim was to evaluate the reasons for the possible delays and propose some solutions to overcome the problems.

## Materials and Methods

The study was conducted in accordance with the principles of the Declaration of Helsinki. With the approval of the local ethics committee, the patients who were newly diagnosed as BC and underwent transurethral resection of bladder tumour (TUR-B) in the last 5 years were retrospectively included in the study. The patients with concomitant malignancy, with a previous diagnosis of BC, and the patients who were diagnosed as COVID-19 during the evaluation were excluded from the study. The patients were divided into 5 groups according to surgery time. Group 1 was composed of newly diagnosed BC patients between March 1, 2016, and March 1, 2017. The patients who were newly diagnosed as BC at the corresponding time periods of the following years were included into Group 2, Group 3, and Group 4, respectively. The last group (Group 5) was composed of patients who were newly diagnosed as BC during the pandemic period which was between March 1, 2020 and March 1, 2021. The reason why we chose this time intervals was related with the restriction period in our country which started on March 1, 2020. During the preceding 12 months, 6 months were under strict restrictions and the other 6 months were under semi-strict restrictions. Even during the strict restriction periods, the patients could reach the hospital with a registered appointment.

The demographic and clinical properties of patients including the age, gender, tumor size, number of tumors, pathological grade, pathological stage, presence of carcinoma in-situ (CIS), presence of lymphovascular invasion (LVI), and presence of variant pathology were noted. The biochemical analysis including urinalysis, urine culture, serum creatinine level, serum prostate specific antigen (for male patients ≥50 years), and the radiological evaluation including urinary system ultrasonography was performed on all patients. Any suspicious patients for bladder cancer (elderly patients with macroscopic or microscopic hematuria) or thepatients who had bladder mass in the urinary system ultrasonography underwent diagnostic cystoscopy. After the visualization of bladder mass, the patients were referred to the anesthesia department for presurgical evaluation. After the approval of anesthesia, the patients were scheduled for TUR-B.

**Figure 1:**
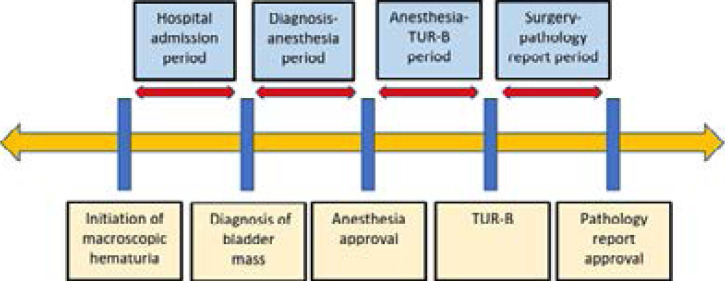
Demonstration of clinical approach and treatment process for patients with bladder cancer. TUR-B: Transurethral resection of bladder tumor

The time schedule of the patients was started from the first day of macroscopic hematuria and ended on the day of the pathology report. As the other lower urinary tract symptoms were not as specific as hematuria for bladder cancer, the beginning of the time schedule was the day of diagnosis for the patients who did not report macroscopic hematuria. The checkpoints of the time schedule were the initiation of macroscopic hematuria, the day of diagnosis of bladder mass, the day of anesthesia approval, the day of TUR-B, and the day of the pathology report. The durations between each checkpoint were analyzed and compared between the groups. The “hospital admission period” was defined as the duration between the first day of macroscopic hematuria and the day of bladder mass diagnosis. The “diagnosis-anesthesia period” was defined as the duration between the day of bladder mass diagnosis and the day of anesthesia approval. The “anesthesia-surgery period” was defined as the duration between the day of anesthesia approval and the day of TUR-B. The “surgery-pathology period” was defined as the duration between the day of TUR-B and the day of pathology report approval. (Figure 1)

## Statistical Analysis

The statistical analysis of the variables was performed by SPSS.21.0 version. The continuous variables of the study were summarized as means and standard deviations, whereas the categorical variables of the study were summarized as frequencies and percentages. The categorical variables of the study groups were compared by Pearson's chi-squared test. The subgroup analysis of the continuous variables was performed by ANOVA test. The p value <0.05 was considered to be significant.

## Results

A total of 303 patients were included in the study. There were 56 patients in Group 1, 60 patients in Group 2, 61 patients in Group 3, 68 patients in Group 4, and 58 patients in Group 5. The mean age of the patients was 66.3±10.6 in Group 1, 64.6±15.7 in Group 2, 68.3±10.4 in Group 3, 69.0±10.6 in Group 4, and 67.0±10.1 in Group 5. (p=0.260) The gender distribution was similar between the groups. (p=0.756) (Table 1) The most frequent symptom of the patients was macroscopic hematuria forming 73.6% of all patients with BC. Other symptoms were irritative symptoms, obstructive symptoms, and renal pain. Bladder cancer was incidentally diagnosed in 18(5.9) patients. When we evaluated the groups according to primary symptom, we observed no difference between the pre-pandemic and pandemic period. (0.998) (Table 2)

**Table 1:** Comparison of the clinicopathological and demographic properties of the patients with bladder cancer before and after the outbreak of SARS COV-2. PUNLMP: Papillary urothelial neoplasm of low malignant potential CIS: Carcinoma in situ, TCC: Transitional cell carcinoma, LV: Lymphovascular

	Group 1 (2016–2017)	Group 2 (2017–2018)	Group 3 (2018–2019)	Group 4 (2019–2020)	Group 5 (2020–2021)	*P* value
Number of patients (n)	56	60	61	68	58	

Age (year)	66.3±10.6	64.6±15.7	68.3±10.4	69.0±10.6	67.0±10.1	*0.260*

Gender						
Male (%)	52(92.8)	52(86.7)	55(90.2)	61(89.7)	54(93.1)	*0.756*
Female (%)	4(7.2)	8(13.3)	6(9.8)	7(10.3)	4(6.9)	

Hydronephrosis						
Yes (%)	5(8.9)	10(16.7)	5(8.2)	13(19.1)	13(22.4)	*0.121*
No (%)	51(91.1)	50(83.3)	56(91.8)	55(80.9)	45(77.6)	

Tumor size						
<3cm.	19(33.9)	20(33.3)	20(34.4)	26(38.2)	23(39.7)	*0.905*
≥3cm.	37(66.1)	40(66.7)	41(65.6)	42(61.8)	35(60.3)	

Number of tumors						
1	41(73.2)	40(66.7)	39(63.9)	34(50.0)	42(72.4)	*0.158*
1–7	15(26.8)	19(31.7)	20(32.8)	32(47.1)	15(25.9)	
>7	-	1(1.6)	2(3.3)	2(2.9)	1(1.7)	

Pathology						
PUNLMP (%)	1(1.8)	-	-	-	-	
CIS (%)	-	-	-	-	1(1.7)	
Ta Low Grade (%)	13(23.2)	15(25.0)	20(32.8)	24(35.3)	23((39.7)	
Ta High Grade (%)	1(1.8)	3(5.0)	5(8.2)	3(4.4)	1(1.7)	*0.239*
T1 Low Grade (%)	10(17.9)	8(13.3)	9(14.8)	4(5.9)	4(6.9)	
T1 High Grade (%)	24(42.8)	19(31.7)	13(21.3)	24(35.3)	17(29.3)	
T2 High Grade (%)	5(8.9)	14(23.3)	14(22.9)	12(17.6)	11(19.0)	
Non-TCC (%)	2(3.6)	1(1.7)	-	1(1.5)	1(1.7)	

Presence of CIS						
Yes (%)	-	3(5.0)	3(4.9)	2(2.9)	3(5.2)	*0.524*
No (%)	56(100)	57(95.0)	58(95.1)	66(97.1)	55(94.8)	

Presence of LV invasion						
Yes (%)	39(69.6)	38(63.3)	36(59.0)	39(57.4)	29(50.0)	
No (%)	17(30.4)	22(37.7)	25(41.0)	29(42.6)	29(50.0)	0.280

Presence of variant pathology						
Yes (%)	2(3.6)	3(5.0)	4(6.6)	8(11.8)	7(12.1)	0.282
No (%)	54(96.4)	57(95.0)	57(93.4)	60(88.2)	51(87.9)	

(*) The statistical difference was between group 4 and 5 compared to groups 1,2and3

(§) The statistical difference was between group 5 and other groups.

($) The difference between was group 1–5, group 2–3, group 2–4, group 2–5, group 1–3, group 2–3, group 4-2, group 5-1, group 5-2 and, group 5-3.

**Table 2:** Comparison of the primary symptoms according to the admission of the patients with bladder cancer to the hospital before and after the outbreak of SARS COV-2. LUTS: Lower urinary tract symptoms

	Group 1 (2016–2017) n=56	Group 2 (2017–2018) n=60	Group 3 (2018–2019) n=61	Group 4 (2019–2020) n=68	Group 5 (2020–2021) n=58	*P* value
Hematuria	41(73.2)	46(76.7)	44(72.2)	47(69.1)	45(77.6)	*0.998*
Irritative LUTS	7(12.5)	6(10.0)	8(13.1)	7(10.3)	6(10.3)	
Obstructive LUTS	4(7.1)	2(3.3)	4(6.5)	6(8.8)	3(5.2)	
Renal pain	1(1.7)	2(3.3)	2(3.3)	3(4.4)	1(1.7)	
Incidental	3(5.5)	4(6.7)	3(4.9)	5(7.4)	3(5.2)	

The mean hospital admission period ranged between 24.5±32.0 and 38.3±69.1 days before the pandemic period whereas it was 102.5±179.0 days during the pandemic period which was significantly higher.(p=0.002) The diagnosis-anesthesia period which was 16.5±45.4 days during the pandemic period was also significantly higher than the diagnosis-anesthesia period during the pre-pandemic period.(p=0.034) The pre-pandemic anesthesia-surgery period ranged between 13.0±14.3 and 20.6±19.1 days which was 18.3±26.5 days during the pandemic period. (p=0.159) The surgery-pathology report period decreased to 12.4±8.1 days during the pandemic which was significantly higher at the corresponding previous year of the pandemic. (Table 3)

**Table 3:** Comparison of the treatment process before and after the outbreak of SARS COV-2

	Group 1 (2016–2017)	Group 2 (2017–2018)	Group 3 (2018–2019)	Group 4 (2019–2020)	Group 5 (2020–2021)	*P* value
Hospital admission period (day)	38.3±69.1	24.5±32.0	29.5±65.5	32.3±47.6	102.5±179.0	***0.002*** *
Diagnosis - anesthesia period (day)	9.6±10.8	6.8±9.9	6.7±12.6	6.7±13.6	16.5±45.4	***0.034*** §
Anesthesia – surgery period (day)	13.0±14.3	13.8±18.9	18.1±14.5	20.6±19.1	18.3±26.5	*0.159*
Surgery-pathology report period (day)	24.4±15.6	26.3±20.9	16.8±12.9	17.8±9.7	12.4±8.1	***<0.001*** $

* The statistical difference was between group 4 and 5 compared to groups 1,2 and, 3

§ The statistical difference was between group 5 and other groups.

$ The difference between was group 1-5, group 2-3, group 2-4, group 2-5, group 1-3, group 2-3, group 4-2, group 5-1, group 5-2 and, group 5-3.

When we compared the clinicopathologic properties of patients at pre-pandemic and pandemic period, we observed that tumor size, number of tumors, pathological stage, pathological grade, presence of CIS, presence of LVI, and the presence of variant pathology were similar between the groups. (Table 1) The rate of MIBC ranged between 8.9% and 23.3% at the pre-pandemic period which was 19.0% during the pandemic period. (p=0.239)

## Discussion

Bladder cancer is an aggressive disease with an age-standardized mortality rate (per 100,000 person/year) was 3.3 for men and 0.86 for women.^12^ It is a progressive disease that tumor grade, number of tumors, tumor diameter, prior recurrence rate, tumor category, presence of concurrent CIS and chronic inflammation are the predictors of tumor progression. Inflammation is accepted as one of the properties of bladder cancer, and nearly 20% of cancers progress because of chronic inflammation.^13^ Immune system response takes a crucial part in carcinogenesis and it plays also a significant role in the development of tumor metastases and progress. A parallel interaction between inflammation and an immune response was reported during COVİD-19 disease. Proinflammatory mediators including interleukins and tumor necrosis factors were dramatically raised in cytokine storm which could lead to aggravate COVID-19 with respiratory distress and exacerbate cancer progression, increasing the mortality of patients with bladder cancer. Intravesical Bacillus Calmette Guerin (BCG) has long been used as an adjuvant therapy for high-risk NMIBC to prevent recurrence and progression thanks to its' immune response. De Vrieze et al. reported that patients who were vaccinated against tuberculosis could fight Covid-19 better compared to the unvaccinated group. For this reason, BCG vaccination could stimulate T and B lymphocytes response to deal with COVID-19 virus easily.^14^ This also reaffirms the role of the innate immune system that can develop memory and could play a vital role against bladder cancer and viral infections.

Predictors of BC that have importance after the diagnosis. On the other hand, delayed diagnosis is also an important variable for the progression and prognosis of bladder cancer. Nearly 80% of bladder cancer is non-muscle invasive bladder cancer during the diagnosis but the delay on the diagnosis may change this proportion.9 Fahmy et al reported that a median two-week delay in the duration betweethe onset of complaint and the first referral to GP lead 5% increase of stages pT2-4. In the same study, authors also reported that the 3 weeks delay to the TUR-B caused a 40% increase in the gross hematuria and a 5% decrease in the rate of tumors smaller than 2 cm.^15^ COVID-19 pandemic period caused a significant risk for hospital admission delays and the standard outpatient mechanisms were interrupted due to hospital management changes. Patients with severe diseases like BC might be adversely affected during this unexpected time period.

Contrary to other health systems, the patients in our country may directly get contact with the specialist without any need for GP consultation. For this reason, we defined the hospital admission period as the time interval between the first time of hematuria and the first admission to the hospital. The mean hospital admission time for BC patients ranged between 24–38 days in the last 4 years before the pandemic period which increased significantly to 102 days at the pandemic period. This was an absolute finding that the pandemic period caused a significant delay in the hospital admission period of patients with BC. We believe that the government's strict restrictions, hospitals' preventive managements, and patient's anxiety for disease transmission were the major reasons for this behavioral change. Patients might prefer to postpone the hospital visits until they had significant symptoms. Even the older patients who are the major candidates of BC might hesitate to visit hospitals and stay at home to prevent possible contamination. Communication with the patients by telemonitoring systems may be a solution for the elderly patients to get in contact with the clinicians without leaving their homes. Boehm et al showed that 63.2% of the urology patients were eligible for telemedicine and 84.7% of these patients preferred telemedical consultation during the pandemic period and they concluded that telemedicine was a reliable system during strict restrictions.^16^ The clinicians may use a telemedicine system during restriction periods to prevent the possible delay of patient admission. During their conversation, the clinicians can perform triage for patients and invite them to the hospital for further evaluation.

The primary adverse effect of the pandemic period might be related to the number of newly diagnosed BC patients. In a Chinese study, Yang et al reported that a total of 180 patients in 2019 and 155 patients in 2020 were diagnosed as BC in their clinic which was not a clinically significant decrease. The authors concluded that even during the pandemic period, patients seek help if they had symptoms like hematuria.^17^ In a similar study from China, Li et al reported that the diagnosis rate of BC among the total urologic cancers were similar before and during the pandemic period and the number of BC diagnoses decreased 9.3% during the pandemic period.^18^ In another study comparing the pathologic specimens during the pre-pandemic and pandemic period, the authors reported a 66% decrease in the number of BC specimens during the 9 weeks of the pandemic period compared to the corresponding previous 2 years.^19^ In a study from Italy, the authors reported 43.6% decrease in the diagnosis of BC during pandemic period.^20^ In another study from Italy, Ferro et al. reported that the time to treatment during the COVID-19 pandemic was prolonged when compared to times prior to pandemic (65 vs. 52 days).^21^ The annual mean number of newly diagnosed patients in our study ranged between 56 and 68 in the corresponding previous 4 years and it was 58 patients during the pandemic time. This data showed that the pandemic period did not affect the number of newly diagnosed BC in our hospital.

After the diagnosis of BC, comes the TUR-B. Oderga et al reported that the rate of TUR-B decreased 49% during the first month of the pandemic period. Although the study showed a significant decrease at the rate of TUR-B, the authors documented the change in surgical numbers at the first month of the pandemic in which the restrictions were at the maximum level.^22^ The data of a limited time within the maximum restriction period might be insufficient to document the effect of the COVID-19 period on oncological patients. For this reason, we evaluated the number of TUR-B surgeries during the 1-year period of pandemic and found no difference compared to the corresponding previous 4 years.

The time interval between the diagnosis and the approval for anesthesia is the second step for the treatment of BC. The prolongation of this period also prolongs the surgery of the patients. Our study showed that the time between the diagnosis and anesthesia approval significantly increased during the pandemic period. According to our knowledge, this was the first study in the literature evaluating the effect of the COVID-19 period on the diagnosis-anesthesia interval. We think that the reason for this delay was related to the conservative precautions of our hospital management. Due to the limitation of daily appointments in the anesthesia outpatient clinic, a very limited number of patients were evaluated in daily practice. This might increase the anesthesia approval time of the patients. Besides this, most of the anesthesiologists had to deal with the corona patients in the intensive care unit which adversely affected the routine processes in the anesthesiology department. We believe that inter-disciplinary communication during the pandemic period may be effective to avoid the delay at this step of evaluation. Patients with BC may be taken into special consideration for anesthesiology approval.

The duration between the anesthesia approval to the day of TUR-B during the pandemic period was not different from the pre-pandemic years. This data showed that our clinical approach to newly diagnosed BC patients did not change during the pandemic period. In a study evaluating the behavior of uro-oncologists, Wittman et al showed that all but one uro-oncologist delayed some or all their oncologic surgeries during the pandemic period.^23^ As we proposed that the delay in the uro-oncologic surgeries could lead to unfavorable oncologic and functional outcomes, we tried to perform the oncological surgeries as soon as possible. This might be related to the suitability of our operation theaters during the pandemic period and the availability of our surgical team. On the other hand, the pandemic period might cause a significant delay in TUR-B in high volume or unsuitable centers.

The time interval between the TUR-B and the pathology report day is another important period for the treatment of BC. The decision of further treatment protocols or the necessity of re-TUR surgery was decided according to the pathology report. Any delay in this step will also interfere with the further treatment of BC. Surprisingly, we observed a significant shortening of the time for pathology reporting during the pandemic period. As the number of pathologists did not chance for the last 5 years, we believe that the decrease in the number of surgical procedures other than oncologic surgeries led the pathologists to evaluate a smaller number of pathology specimens. This might be the explanation for the faster pathology reporting period.

According to our knowledge, our study was the first study comparing the macroscopic tumor characteristics of the BC patients before four years and during the pandemic period. Our results showed no differences between the pre-pandemic and pandemic period in terms of the tumor size and the number of tumors in BC patients. The presence of hydronephrosis which might be a finding of invasive BC was also similar between the groups. Tumor stage and grade are the main predictors of disease progression and cancer-specific mortality for BC. The delay in the diagnosis of BC during the pandemic period might lead to stage progression which might have a significant clinical effect. On the other hand, we also did not find a difference between the pre-pandemic period and pandemic period in terms of pathological stage and grade of BC patients. Similar to our findings, Yang et al also reported that the rate of NMIBC and MIBC were similar at pre-pandemic and pandemic periods.^17^ Other pathological prognostic factors like; the presence of CIS, the presence of LV invasion, and the presence of variant pathology were similar at the BC patients who were diagnosed at pre-pandemic and pandemic period. Long term follows up of the BC patients that were diagnosed during the pandemic period will show us the possible bad consequences of this period on the patients.

## Conclusion

As a conclusion, the pandemic period caused some delays in the treatment of BC patients. The hospital admission time and the diagnosis-anesthesia approval time significantly increased whereas the anesthesia reporting time significantly decreased. On the other hand, the number of newly diagnosed BC patients and the histopathologic properties of these patients did not change during the pandemic time. Telemonitoring systems for patients and inter-disciplinary communications may be useful to prevent the possible diagnostic and treatment delays for newly diagnosed BC patients. The follow-up of these patients will more significantly demonstrate the effect of the pandemic period on BC patients.

## Data Availability

Not applicable.
